# Do we need to evaluate diastolic blood pressure in patients with suspected orthostatic hypotension?

**DOI:** 10.1007/s10286-017-0409-7

**Published:** 2017-02-27

**Authors:** Artur Fedorowski, Viktor Hamrefors, Richard Sutton, J. Gert van Dijk, Roy Freeman, Jacques WM Lenders, Wouter Wieling

**Affiliations:** 1 Department of Clinical Sciences, Faculty of Medicine, Clinical Research Center, Lund University, Malmö, Sweden; 2 Department of Cardiology, Skåne University Hospital, Inga Marie Nilssons gata 46, 205 02 Malmö, Sweden; 3 Department of Medical Imaging and Physiology, Skåne University Hospital, Malmö, Sweden; 4 National Heart & Lung Institute, Imperial College, London, UK; 5 Department of Neurology, Leiden University Medical Centre, Leiden, The Netherlands; 6 Department of Neurology, Harvard Medical School, Beth Israel Deaconess Medical Center, Boston, USA; 7 Department of Internal Medicine, Radboud Medical Centre, Nijmegen, The Netherlands; 8 Department of Medicine III, Technical University Dresden, Dresden, Germany; 9 Department of Internal Medicine, Academic Medical Centre, University of Amsterdam, Amsterdam, The Netherlands

**Keywords:** Orthostatic hypotension, Blood pressure, Diastolic, Syncope, Orthostatic intolerance

## Abstract

**Purpose:**

The contribution of diastolic blood pressure measurement to the diagnosis of classical orthostatic hypotension is not known. We aimed to explore the prevalence of isolated systolic and diastolic orthostatic hypotension components in patients with syncope and orthostatic intolerance.

**Methods:**

A total of 1520 patients aged >15 years with suspected syncope and/or symptoms of orthostatic intolerance were investigated in a tertiary center using tilt-table testing and continuous non-invasive blood pressure monitoring. Classical orthostatic hypotension was defined as a decline in systolic blood pressure ≥20 mmHg and/or diastolic blood pressure ≥10 mmHg at 3 min of tilt test. The prevalence of upright systolic blood pressure <90 mmHg and its overlap with isolated diastolic orthostatic hypotension was also assessed.

**Results:**

One hundred eighty-six patients (12.2%) met current diagnostic criteria for classical orthostatic hypotension. Of these, 176 patients (94.6%) met the systolic criterion and 102 patients (54.8%) met the diastolic criterion. Ninety-two patients (49.5%) met both systolic and diastolic criteria, whereas ten patients (5.4%) met the diastolic criterion alone. Of these, three had systolic blood pressure <90 mmHg during tilt test and were diagnosed with orthostatic hypotension on the grounds of low standing blood pressure. Based on patient history and ancillary test results, causes of orthostatic intolerance and syncope other than orthostatic hypotension were present in the remaining seven patients.

**Conclusions:**

An abnormal orthostatic fall in diastolic blood pressure without an abnormal fall in systolic blood pressure is rare among patients with syncope and orthostatic intolerance. Approximately 95% of patients with classical orthostatic hypotension can be identified by systolic criterion alone.

## Introduction

Classical orthostatic hypotension (OH) is defined as a sustained reduction of systolic blood pressure (SBP) of at least 20 mmHg and/or diastolic BP (DBP) of 10 mmHg within 3 min of standing or head-up tilt to at least 60°. These expert-based criteria were originally defined in 1996 in a consensus statement endorsed by the American Autonomic Society and the American Academy of Neurology [1, 11], and were later adopted by the European Society of Cardiology (ESC) syncope guidelines [17]. Although the definition of OH includes separate criteria for SBP and DBP changes, in daily practice the diastolic criterion seems to be rarely used to diagnose OH.

We aimed to examine the contribution of the SBP and DBP criteria, applied separately and in combination, to the detection of OH. We hypothesized that only a minority of OH patients would be identified by DBP criteria alone. Accordingly, we assessed proportions of patients who met the systolic and diastolic OH criteria from a large database of patients investigated for unexplained syncope and/or orthostatic intolerance.

## Materials and methods

### Study setting and population

Between September 2008 and May 2016, a total of 1533 patients aged >15 years with suspected syncope and/or symptoms of orthostatic intolerance unexplained by initial evaluation [17] were investigated at the Syncope Unit of Skåne University Hospital, Malmö, Sweden. Patients were recruited through referrals from primary care and from hospitals in the southern region of Sweden. Prior to investigation at the Syncope Unit, additional tests including exercise and continuous 24-h ECG, external and implantable event recorder, echocardiography, coronary angiography, brain imaging and EEG were performed to exclude cardiac and neurological causes of symptoms. We excluded eight patients with scleroderma and five with supine SBP <90 mmHg. Patients with scleroderma had unreliable readings of finger artery blood pressure, usually falsely low, whereas patients with supine SBP <90 mmHg were hypotensive at baseline and we decided to exclude them for the clarity of results interpretation. This yielded 1520 patients with complete data eligible for the study; of these, 1382 (91%) had a history of suspected syncope and 1089 (72%) reported orthostatic intolerance. The study protocol was approved by the Regional Ethical Review Board in Lund, Sweden (ref. no. 82/2008), and all study participants gave their written informed consent. Written consent on behalf of minors was obtained from parents.

### Examination protocol

The patients were asked to take their regular medication and fast for 2 h before the test, but they were allowed to drink water at will. Prior to examination, the patients were asked to complete a questionnaire which explored past medical history, duration, frequency and features of syncope-related symptoms, smoking status and current pharmacological treatment. After supine rest for at least 10 min, a standardized 70° head-up tilt test (HUT) was performed until syncope/presyncope or pronounced symptoms of orthostatic intolerance occurred, or for a maximum of 20 min, followed by optional nitroglycerin provocation according to the Italian protocol [3]. Prior to HUT, carotid sinus massage was performed in patients aged ≥40 years according to the Newcastle protocol [20], and the Valsalva maneuver completed HUT [13]. Beat-to-beat blood pressure (BP) and electrocardiogram (ECG) were continuously monitored using a validated noninvasive photoplethysmographic method (Nexfin monitor; BMEYE, Amsterdam, Netherlands) [4], and subsequently analyzed offline using a dedicated program provided by the manufacturer.

### Data analysis

Blood pressure and heart rate (HR) in the supine position 1 min prior to HUT and at 3 min of HUT were calculated as an average of a 30-s period and recorded in the database. The predefined point for the second hemodynamic assessment at 3 min of HUT was selected to comply with the current definition of classical orthostatic hypotension [11]. If the syncope occurred within the 3-min HUT period, the last 30-s period before the onset of prodromal symptoms, profound hypotension and/or bradycardia was analyzed and averaged. Systolic OH was defined as SBP decline ≥20 mmHg, and diastolic OH as DBP decline ≥10 mmHg within 3 min of HUT [11]. In addition, we also assessed the prevalence of upright SBP <90 mmHg [17], as stated in the current ESC guidelines, and SBP decline ≥30 mmHg in patients with supine SBP ≥160 mmHg, as proposed by a previous study of OH in hypertensive and normotensive patients [5, 29].

### Statistical analyses

The main characteristics of the study population are presented as mean and standard deviation for continuous variables and as percentages for categorical variables. Group differences in continuous variables were compared using analysis of variance (ANOVA), and dichotomous variables were compared using Pearson’s chi-square test. All analyses were performed using IBM SPSS Statistics version 23 software (IBM Corp., Armonk, NY, USA). All tests were two-sided, if applicable, wherein *p* < 0.05 was considered statistically significant.

## Results

**Fig. 1:**
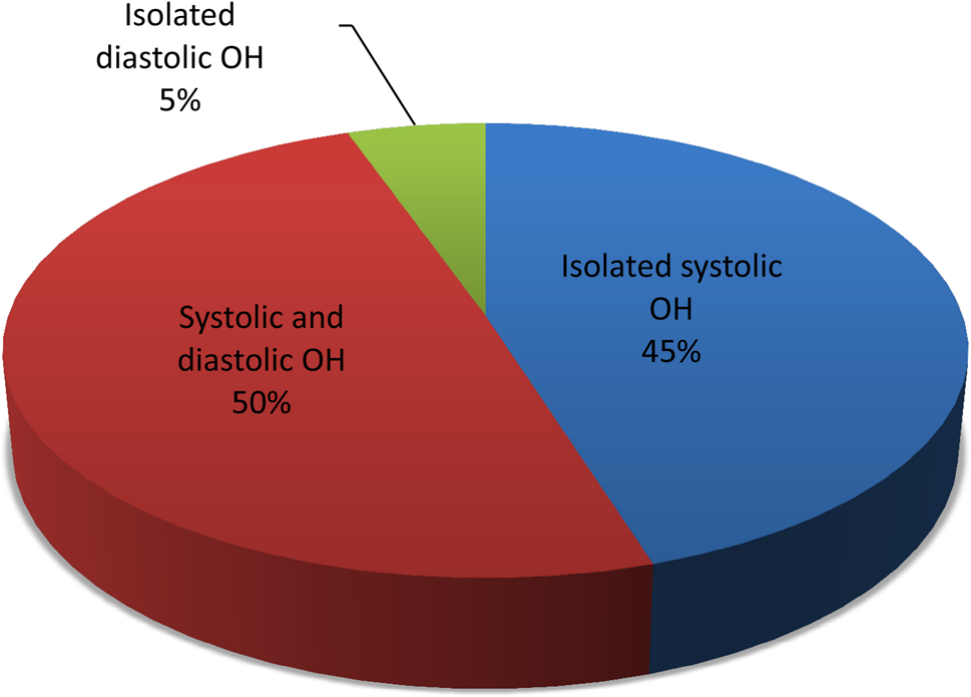
Proportions of patients (*n* = 186) diagnosed with classical orthostatic hypotension (OH) according to current consensus criteria stratified into isolated systolic OH (*n* = 84), systolic and diastolic OH (*n* = 92), and isolated diastolic OH (*n* = 10). *SBP* systolic blood pressure, *DBP* diastolic blood pressure. Systolic OH = orthostatic SBP decline ≥20 mmHg; diastolic OH = orthostatic DBP decline ≥10 mmHg

The characteristics of the study population are presented in Table 1. There was a slight predominance of women, and the mean age was 53 years. One-third of patients reported a history of hypertension and current antihypertensive treatment. Among the total 1520 patients, 186 (12.2%) met the current diagnostic criteria of OH using combined cutoff values for either SBP or DBP. Of these, 176/186 (94.6%) met the systolic criterion. and 102/186 (54.8%) met the diastolic OH criterion. A total of 92/186 (49.5%) patients met both systolic and diastolic criteria, whereas 84/186 (45.2%) met only the systolic criterion and 10/186 (5.4%) met only the diastolic criterion (Fig. 1). Thus, of the total of 186 patients with OH according to the current consensus criteria, only one of 19 was classified as having OH on the grounds of isolated DBP decrease.

**Table 1:** Characteristics of study participants. Data are presented as number and percentage or mean and standard deviation

Characteristic	All (*n* = 1520)	No OH (*n* = 1334)	ΔSBP ≤ −20 mmHg (*n* = 176)	*p* value vs. no OH	ΔDBP ≤ −10 mmHg only* (*n* = 10)	*p* value vs. no OH
Age (years)	53 ± 21	51 ± 21	68 ± 15	<0.001	58 ± 23	0.31
Sex (male)	602 (40)	495 (37)	103 (59)	<0.001	4 (40)	0.85
BMI (kg/m^2^)	25 ± 5	25 ± 5	25 ± 4	0.17	24 ± 3	0.47
SBP supine (mmHg)	132 ± 22	130 ± 21	146 ± 26	<0.001	130 ± 31	0.98
DBP supine (mmHg)	72 ± 10	71 ± 9	76 ± 12	<0.001	76 ± 12	0.11
Heart rate supine (beats/min)	70 ± 12	70 ± 12	70 ± 12	0.47	71 ± 14	0.89
SBP 3-min HUT (mmHg)	130 ± 24	133 ± 23	109 ± 27	<0.001	120 ± 40	0.62
DBP 3-min HUT (mmHg)	76 ± 12	78 ± 11	65 ± 14	<0.001	63 ± 12	<0.001
Heart rate 3-min HUT (beats/min)	81 ± 16	80 ± 17	79 ± 17	0.33	83 ± 14	0.66
History of hypertension	450 (30)	368 (28)	79 (45)	<0.001	3 (30)	0.71
Current antihypertensive treatment	529 (35)	437 (33)	88 (50)	<0.001	4 (40)	0.47
History of diabetes mellitus	104 (7)	87 (7)	16 (9)	0.43	1 (10)	0.88
History of coronary heart disease (AMI/CABG/PCI)	109 (7)	85 (6)	24 (14)	<0.001	0 (0)	0.43
Current smoking	193 (13)	172 (13)	20 (12)	0.50	1 (10)	0.98

*BMI* body-mass index, *OH* orthostatic hypotension, *SBP* systolic blood pressure, *DBP* diastolic blood pressure, *HUT* head-up tilt test, *AMI* acute myocardial infarction, *CABG* coronary artery bypass graft, *PCI* percutaneous coronary intervention

* Excluding delta SBP ≤ −20 mmHg

Patients with systolic OH were older and more likely men, had higher supine SBP and DBP, and a higher proportion of hypertension, antihypertensive treatment, and manifest coronary disease compared with non-OH patients (Table 1, *p* ≤ 0.001 for all comparisons). One hundred fifty-nine patients with systolic OH (90.3%) were diagnosed with syncope due to OH by the clinicians performing the tests, whereas in the remaining 17 patients (9.7%), systolic OH was detected but not found to be decisive for the syncope etiology.

In the supine SBP range equal to or above 160 mmHg (*n* = 170/1520, 11.2%), a total of 49 patients met the current systolic criterion of SBP decline ≥20 mmHg; of these, 34 had SBP decline ≥30 mmHg (*p* < 0.001 for difference between groups). The proportions of patients classified with abnormal orthostatic BP according to different criteria ranged from 11.4% for modified systolic criteria including higher systolic threshold in more severe hypertension, to 12.4% for systolic OH criterion plus standing SBP <90 mmHg but without isolated diastolic OH (Table 2).

**Table 2:** Proportions of subjects with abnormal orthostatic blood pressure changes according to different criteria in a population of patients (*n* = 1520) with unexplained syncope and/or orthostatic intolerance

Diagnostic criteria	*n* (%)	*n* (difference)
ΔSBP ≤ −20 mmHg and/or ΔDBP ≤ −10 mmHg*	186 (12.2)	Reference
ΔSBP ≤ −20 mmHg only	176 (11.6)	−10
ΔSBP ≤ −20 mmHg and/or 3-min HUT SBP <90 mmHg^a^	188 (12.4)	+2
ΔSBP ≤ −20 mmHg if supine SBP <160 mmHg or ΔSBP ≤ −30 mmHg if supine SBP ≥160 mmHg or 3-min HUT SBP < 90mmHg^a^	173 (11.4)	−13

*SBP* systolic blood pressure, *DBP* diastolic blood pressure, *HUT* head-up tilt test

* Orthostatic hypotension definition according to the current Autonomic Societies Consensus (2011)

^a^Definition of orthostatic hypotension according to the current European Society of Cardiology guidelines for active standing test (2009)

The detailed hemodynamic parameters of the ten patients with isolated diastolic OH are shown in Table 3. The mean ΔDBP was −14 ± 7 mmHg (range −27 to −10 mmHg). None of the basic biometric and clinical parameters including age, sex, BMI, supine SBP, DBP and heart rate, history of hypertension, coronary disease or diabetes, and smoking differed significantly from the rest of the cohort. Three patients with isolated diastolic OH had upright SBP below 90 mmHg after 3-min HUT (#1–3), and were diagnosed with syncope due to OH on the grounds of patient history and test results. In two of these patients, symptoms of orthostatic intolerance were considered to be a side effect of antihypertensive drugs. One of the seven remaining patients with isolated diastolic OH, a 67-year-old woman (#10), had a very high SBP above 200 mmHg and was diagnosed with vasovagal syncope (VVS) after nitroglycerine challenge and reproduction of previous attacks. The six remaining patients (#4–9) were predominantly younger/middle-aged women (5/6) without antihypertensive treatment who were normotensive on standing (SBP, 96–137 mmHg), and two had 3-min HUT SBP below 120 mmHg (Table 3). These patients were diagnosed with VVS after reproducing syncope during HUT. The only male patient in this group, an 87-year old (#6), was diagnosed with syncope due to carotid sinus syndrome.

**Table 3:** Hemodynamic data of ten patients with isolated diastolic orthostatic hypotension recorded in the supine position and after 3-min head-up tilt test (HUT) with the most likely syncope etiology

Patient Gender/age (years)/AHT	BP supine (mmHg)	BP 3-min HUT (mmHg)	ΔDBP (mmHg)	Syncope etiology*
M/84/yes	100/62	82/49	−13	OH
M/82/yes	101/62	83/52	−10	OH
M/41/no	103/65	89/55	−10	OH
F/34/no	110/67	96/56	−11	VVS
F/22/no	125/72	109/56	−16	VVS
M/86/yes	134/76	122/62	−14	CSS
F/35/no	135/83	137/56	−27	VVS
F/63/no	143/89	126/78	−11	VVS
F/66/no	150/90	134/79	−11	VVS
F/67/yes	201/92	217/81	−11	VVS

*M* male, *F* female, *y* years, *BP* blood pressure, *DBP* diastolic blood pressure, *AHT* antihypertensive treatment, *OH* orthostatic hypotension, *VVS* vasovagal syncope

* The investigator determined the most likely syncope etiology based on patient’s history, results of additional tests and HUT

Patients with supine SBP ≥160 mmHg demonstrated more pronounced changes in SBP, DBP and heart rate after 3-min HUT compared with supine SBP <160 mmHg (Table 4). However, among those who met the systolic OH criterion, there was a significant difference between the two groups only in ΔSBP (−45 ± 24 vs. −34 ± 13 mmHg; *p* < 0.001), i.e. those with systolic OH who had higher supine SBP demonstrated a more pronounced SBP decline during HUT.

**Table 4:** Hemodynamic changes after 3-min head-up tilt test in the cohort of 1520 patients with history of syncope and/or symptoms of orthostatic intolerance stratified according to supine blood pressure below vs. equal to or above 160 mmHg presented as mean ± SD

Hemodynamic parameter	All patients			3-min HUT ΔSBP ≤ −20 mmHg		
	All patients *n* = 1520	Supine SBP <160 mmHg *n* = 1350	Supine SBP ≥160 mmHg *n* = 170	All patients *n* = 176	Supine SBP <160 mmHg *n* = 127	Supine SBP ≥160 mmHg *n* = 49
ΔSBP (mmHg)	−2 ± 17	−1 ± 15	−12 ± 27*	−37 ± 18	−34 ± 13	−45 ± 24*
ΔDBP (mmHg)	+5 ± 9	+5 ± 8	+1 ± 13*	−11 ± 11	−10 ± 9	−12 ± 15
Δ Heart Rate (beats/min)	10 ± 11	11 ± 11	7 ± 9*	+10 ± 11	+10 ± 12	+9 ± 8

*SBP* systolic blood pressure, *DBP* diastolic blood pressure, *HUT* head-up tilt test

* *p* < 0.001 for difference between the groups (supine SBP below vs. equal to or above 160 mmHg)

## Discussion

We report here that an abnormal decrease in diastolic blood pressure without an abnormal decrease in systolic pressure is very rare among patients investigated for suspected syncope and orthostatic intolerance. The overwhelming majority of patients with OH can be identified by a systolic criterion. Moreover, the isolated DBP decrease seems not to be decisive for the final diagnosis of syncope and the management of patients.

### Clinical implications of OH criteria

Current diagnostic criteria of OH include both systolic and diastolic cutoff [11]. These criteria are expert-based. In 1996, a consensus committee of the American Autonomic Society and the American Academy of Neurology met to discuss the etiological criteria of OH and to determine how OH should be diagnosed [1]. Prior to this consensus, investigators and clinicians used varying numbers to denote the presence of OH, creating confusion. The combined clinical wisdom of this group of experts (*n* = 13) proclaimed that a 20-mmHg systolic and/or a 10-mmHg diastolic decline from lying to standing within 3 min of standing should be the standard. The 1996 criteria were primarily based on a study of 92 male and female normal subjects aged 17–61 years [24, 25], as epidemiological data were unavailable at that time. A later study by Fedorowski et al. in a population-based cohort of 924 subjects confirmed that the older 20/10-mmHg standard for the definition of OH was an excellent cutoff for normotensive persons [5]. However, in subjects with resting SBP above 160 mmHg, a fall of 30 mmHg should be used. Moreover, only 10% of subjects who met the diagnostic criteria of OH (9/88) did so on the grounds of isolated diastolic OH [5].

In clinical practice, the diastolic criterion is often ignored. This may have several reasons: First, the absolute magnitude of changes in SBP is larger than that of DBP, and is thus much easier to measure. Second, the accuracy of BP measurements may vary by around 5 mmHg [19] due to blood pressure oscillations and measurement imprecision—an amount that is half of the diagnostic threshold for DBP, engendering greater confidence in the change in SBP. Finally, an abnormal fall in DBP with a minor or no fall in SBP will increase pulse pressure. Since the main determinants of brain blood flow are the absolute level of arterial pressure and the pulse pressure [27], an isolated fall in diastolic pressure is not likely to induce significant hypoperfusion of the brain. It has been shown that symptoms of orthostatic intolerance such as dizziness or (pre-)syncope are strongly dependent on SBP and not on DBP decline [23].

Consequently, for a clinically relevant diagnosis of OH in symptomatic patients, the systolic criteria seem to be sufficient. They will identify approximately 95% of subjects with OH based on the current consensus and the vast majority of abnormal orthostatic BP responses. Adding the absolute SBP threshold of below 90 mmHg on standing may further expand the systolic OH criteria, with the total number of cases being almost the same as for the combined systolic-diastolic criteria. The value of additional systolic criterion may be justified by the fact that symptomatic patients with hypotension (SBP <90 mmHg) on standing may require clinical intervention, and their identification could be important. This is of relevance in individuals with low SBP where the current OH criteria may miss a clinically significant fall in cerebral perfusion due to a narrow range of BP fall.

In addition, for resting SBP above 160 mmHg, a higher diagnostic threshold of SBP decline ≥30 mmHg, as previously proposed, could be considered [5, 29]. In patients with severe hypertension, the natural fluctuations of BP are greater [16], as was also shown in our study. Moreover, pronounced BP swings that lead to an apparent normalization of supine hypertension on standing are very common in autonomic failure [26]. Thus, the specificity of OH diagnosis in the more severe hypertension might be improved by a higher diagnostic SBP threshold to avoid falsely positive cases due to increased BP variability.

### Diastolic BP and neurogenic OH

A diastolic BP decline within 3 min of standing equal to or greater than 10 mmHg on at least three separate occasions has been proposed by Streeten as a characteristic and obligatory sign of neurogenic OH—i.e. severe autonomic failure [25]. Streeten postulated that an absence of significant and consistent diastolic decline would preclude the diagnosis of neurogenic OH. However, he also observed that both neurogenic and non-neurogenic patients presented with significant SBP fall, and practically all these patients could be identified as having OH on the grounds of systolic criterion alone.

From a clinical point of view, cerebral hypoperfusion is the most important aspect of OH that must be addressed and, in highly symptomatic patients, treated [21]. It has been previously shown that for both symptom generation and therapy monitoring, the systolic rather than the diastolic (alone or in combination with systolic) hypotension is the finding that carries the clinical importance [23]. As for the diagnostic utility of DBP assessment, patients with suspected neurogenic OH are usually referred to and evaluated by experts in tertiary centers with access to reliable diagnostic methods [10, 12, 13]. Thus, the role of DBP in diagnosis of neurogenic OH is uncertain today and should be elucidated in well-designed studies performed in centers with experience in this condition.

### Diastolic OH and long-term prognosis

Even though diastolic decline in BP during orthostasis may be less relevant in the clinical diagnosis of OH, its potential impact on long-term prognosis must be borne in mind. Orthostatic hypotension has been consistently associated with increased mortality and incidence of cardiovascular disease in large population-based prospective studies [2, 22]. Although a significant SBP decrease on standing demonstrates a similar risk as combined OH criteria [6, 7], in several studies an independent association between diastolic (and often asymptomatic) OH and higher incidence of myocardial infarction has been observed [8, 9, 15]. Moreover, a greater decline in DBP, i.e. equal or more than 20 mmHg, has been linked with higher mortality in older patients [14]. We propose that in future epidemiological studies, isolated diastolic OH should continue to be assessed to clarify this point.

## Study strengths and limitations

The current OH diagnostic criteria are based on expert opinion and tests performed in small groups of patients and healthy individuals. The predominant techniques used at that time were active standing test and intermittent BP measurement using auscultatory or oscillometric methods [18]. The present study is based on a large sample of symptomatic individuals, a 70° head-up tilt test, which is a standardized passive orthostatic challenge method, and non-invasive continuous photoplethysmographic technology of hemodynamic monitoring. The study design is therefore generalizable to typical syncope and autonomic disorder evaluation laboratories. However, our observations should be verified against similar settings in independent populations, and compared with conventional BP measurements using a sphygmomanometer. In addition, patients with neurogenic OH due to neurodegenerative diseases such as Parkinson’s disease, multiple system atrophy and pure autonomic failure may have been underrepresented in our study populations. Thus, our conclusions should be taken with caution in regard to patients with neurogenic orthostatic hypotension. Further, initial OH was not assessed in this study, and the contribution of isolated DBP fall to the diagnosis of initial OH remains unexplored [28]. Finally, the current definition of OH is centered on measurement results and not on the associated complaints. Thus, we would like to emphasize the possible discrepancy between OH based on the abnormal orthostatic BP response observed during diagnostic tests and its relevance for the patient’s symptoms and the most likely syncope etiology.

## Conclusions

An isolated abnormal orthostatic drop in DBP without a significant fall in SBP is rare among patients with unexplained syncope and orthostatic intolerance. Approximately 95% of patients with classical OH can be identified by systolic criteria alone. Our data imply that the systolic criterion might be used instead of current OH definition based on both systolic and diastolic criteria.
